# The association between working memory, teacher-student relationship, and academic performance in primary school children

**DOI:** 10.3389/fpsyg.2023.1240741

**Published:** 2023-09-22

**Authors:** Simona Sankalaite, Mariëtte Huizinga, Petra Warreyn, Jolien Dewandeleer, Dieter Baeyens

**Affiliations:** ^1^Parenting and Special Education Research Unit, Faculty of Psychology and Educational Sciences, Katholieke Universiteit Leuven, Leuven, Belgium; ^2^Department of Educational and Family Studies, Faculty of Behavioural and Movement Sciences, Vrije Universiteit Amsterdam, Amsterdam, Netherlands; ^3^Department of Experimental Clinical and Health Psychology, Faculty of Psychology and Educational Sciences, Ghent University, Ghent, Belgium

**Keywords:** working memory, teacher-student relationship, academic performance, primary school, cross-lagged panel model

## Abstract

**Introduction:**

Early relationships with teachers play an important role in children’s development and significantly influence students’ cognitive and academic performance. Studies suggest that working memory (WM) is a strong predictor of academic achievement, especially of reading and arithmetic outcomes. The associations between teacher-student relationship (TSR) quality, children’s WM skills and their academic performance have been reported in numerous observational studies. However, the potentially bidirectional and temporal nature of the relationships between these constructs is understudied.

**Methods:**

The purpose of this study was to investigate the relationships between primary school children’s WM and TSR by applying a cross-lagged design and measuring these constructs at three time points throughout the academic year. More exploratively, this study investigated how WM and TSR bidirectionally relate to children’s academic performance.

**Results:**

The findings of this study revealed a temporal relationship between WM and TSR: between WM-related problems in the classroom at baseline and conflict at 3-month follow-up, and between closeness at 3-month follow-up and WM-related problems in the classroom at 5-month follow-up. Moreover, the findings showed a bidirectional relationship between arithmetic performance and WM-related problematic behaviour.

**Discussion:**

This study highlights that relationships between the teacher and students play an important role in supporting students’ cognitive and academic development. Importantly, this study suggests that children with WM problems may benefit from interventions that focus on improving their relationships with teachers. Additionally, the findings propose that interventions targeting WM may also have positive effects on children’s academic performance.

## Introduction

Early relationships with teachers play a vital role in children’s development (Hamre and Pianta, 2001). Teacher-student relationships (TSR) significantly affect students’ cognitive skills and academic performance, especially when the quality of these relationships is high. Furthermore, studies suggest that working memory (WM) is a strong predictor of academic achievement, especially of reading and arithmetic outcomes (e.g., Huizinga et al., 2018; for a review see Cortés Pascual et al., 2019). The associations between the quality of TSR and children’s WM skills have been reported in numerous observational studies (for reviews see Cumming et al., 2019; Koşkulu-Sancar et al., 2023). However, the potentially bidirectional and temporal nature of the relationships between these constructs, as well as how each of these constructs relate to academic performance, remain understudied. Such findings can further inform classroom interventions, teaching strategies and training by providing valuable insights on how each construct evolves and influences each other (for a review see Sankalaite et al., 2021).

### Working memory

Executive function (EF) is an umbrella term describing various cognitive processes that are required to carry out conscious goal-directed behaviour and are especially important in novel and demanding situations, which require a rapid and flexible adjustment of behaviour to the changing demands of the environment (Huizinga et al., 2006; Diamond, 2013). It is well documented that EFs are fundamental for children’s learning, school functioning and academic achievement (e.g., Huizinga et al., 2018; for a review see Cortés Pascual et al., 2019). Core EFs include inhibitory control, cognitive flexibility, and working memory (WM) (Diamond, 2013). However, WM—the ability to temporarily store, update and manipulate information (particularly important for reasoning, decision-making and problem-solving) is the most predictive (out of all EF subcomponents) of academic achievement, especially in domains of reading and mathematics (for a review see Allen et al., 2020; for a meta-analysis see Peng et al., 2016).

Based on the literature, two main models have been proposed to conceptualise WM. Baddeley and Hitch (1974) introduced a three-component model of WM, comprising the central executive and two storage systems: the phonological loop and the visuospatial sketchpad, later supplemented by a fourth component—the episodic buffer (Baddeley, 2000; Baddeley et al., 2011). In contrast, Cowan (1999, 2010) suggested an embedded-process model of WM, which mostly focuses on the underlying cognitive processes occurring when solving a task (e.g., language comprehension, decision-making). Cowan’s model consists of four elements: central executive, long-term memory, activated memory, and the focus of attention. Both of these models broadly distinguish WM into verbal and visuo-spatial components. In Cowan’s model, the distinction is based on their representational formats within the activated memory and the focus of attention while, in Baddeley’s model, as separate stores for verbal and visuospatial information. Given that these two distinct components involve different cognitive processes and pose different cognitive demands, to obtain a more comprehensive understanding of an individual’s WM capacities, they should be assessed independently (Alloway et al., 2006). Both models underscore the complexity of WM, which is important to consider when assessing WM and understanding its significance in learning.

Working memory develops rapidly in preschool (Gómez et al., 2018) laying the groundwork for other EF components (Diamond, 2013). Research suggests that substantial growth in WM can be seen between ages of 4–15 years (Gathercole et al., 2004), fluctuating between more steady and more rapid growth periods. Indeed, the growth rate is not linear, and a particular developmental spurt occurs during early and middle childhood (Alloway et al., 2004), slightly slowing during adolescence (Ahmed et al., 2022). The development of WM mirrors developmental changes in brain structures, such as the maturation of the prefrontal cortex, synaptic pruning and myelination (Kolk and Rakic, 2021). Taking into account results from both behavioural and neuroimaging research, (early and middle) childhood seems to be a period characterised by plasticity, sensitivity, and responsivity to developmental and environmental influences (Zelazo and Carlson, 2012, 2020; McEwen and Morrison, 2013; Thompson and Steinbeis, 2020; Tooley et al., 2021). Intervening during this time in the development can, therefore, have a substantial impact to children’s further cognitive outcomes.

Working memory is essential for learning and academic achievement (Gathercole and Alloway, 2008). It helps children process and understand new information, follow instructions, and remember and apply knowledge (Jaroslawska et al., 2015). For example, when reading, children need to hold the meaning of words or sentences in their WM to comprehend the text. Furthermore, WM capacity is linked to attention and focus (Shipstead et al., 2014). While attention ensures a selection of relevant pieces of information, WM then maintains these basic elements active during processing and ensures a successful arrangement of information (Marchetti, 2010, 2014). This is particularly important in classroom settings where children need to concentrate on tasks and filter out irrelevant information. In addition, WM plays a vital role in problem-solving and reasoning (Swanson and Sachse-Lee, 2001). It allows children to hold multiple pieces of information in mind, manipulate them, and draw connections. For example, when solving math problems, children use WM to hold numbers, remember the steps, and perform mental calculations (Berg, 2008). Finally, WM is involved in language processing and communication (Pauls and Archibald, 2022). It helps children remember and comprehend sentences, follow conversations, and generate coherent responses. Children with weaker WM may struggle with tasks such as remembering and following instructions, organising their thoughts, and expressing themselves clearly. Focussing on improving WM in middle childhood can, therefore, enhance learning outcomes in areas like reading and mathematics, which become increasingly more complex during the primary school years.

It is well-established that WM outcomes can vary depending on the context in which they are measured (e.g., Toplak et al., 2012; Miranda et al., 2015; Veloso and Ty, 2021). Performance tasks, which are conducted in a controlled, stimulus-free environment, may not fully capture the complexities of WM in real-world situations (Toplak et al., 2012). These tasks typically involve presenting participants with a set of stimuli to remember and then asking them to recall and manipulate that information in some way. Behavioural ratings, on the other hand, involve observing WM in the classroom or other real-world contexts and, therefore, may provide a more ecologically valid measure of WM (e.g., Tan et al., 2017). However, behavioural ratings can be subject to several factors that can influence their validity and reliability, such as observer bias or differences in the characteristics of the settings being observed. Therefore, performance tasks and behavioural ratings can complement one another in providing a more complete picture of how WM operates in different contexts.

### Teacher-student relationship

A dynamic systems perspective is a theoretical framework that emphasises the importance of studying complex constructs across contexts and as a whole rather than reducing them to their individual components (Thelen and Smith, 2006). Applied to WM, this perspective highlights the need to focus not only on WM abilities but also on the context in which WM-related strengths and weaknesses are evident, as well as other factors that are at interplay with WM. Previous research suggests that positive social interactions and relationships with both parents (i.e., home setting) and teachers (i.e., classroom context) can promote WM performance (e.g., Vandenbroucke et al., 2017).

It is well-established that early relationships with adults play an important role in children’s social, emotional, cognitive and academic development (Hamre and Pianta, 2001; Immordino-Yang et al., 2019). When applied to social interactions, and, more specifically, to adult-child relationships, a dynamic systems perspective (Thelen and Smith, 2006) highlights that such relationships are complex and non-linear, and are influenced by multiple factors that interact over time. Generally, positive relationships, characterised by praise, responsiveness, encouragement, and scaffolding, can lead to more positive outcomes and influence on WM (e.g., Bernier et al., 2010; Schroeder and Kelley, 2010; Mermelshtine, 2017). Furthermore, favourable children’s behaviour or successful academic performance can, in turn, lead to positive reinforcement from parents or increase in parental involvement in child’s education upon observing success (Ðurišić and Bunijevac, 2017). On the other hand, negative relationships, characterised by punishment, control and criticism, can adversely impact WM performance (e.g., Rhoades et al., 2011; for reviews see Fay-Stammbach et al., 2014; Valcan et al., 2017). When children face academic struggles, it can lead to increased parental pressure, which can exacerbate anxiety and reduce intrinsic motivation in the child (Pomerantz et al., 2007). Parent-child relationships are one of the primary contexts for children’s cognitive development and promoting WM (Hughes, 2011). However, as children grow, their social context expands beyond the home environment and immediate family members, and relationships with peers, as well as teachers start to play a role in their cognitive, emotional, and social development (Jing et al., 2020).

Given that primary school-age children spend a significant amount of time in school, the focus of recent research has expanded from parent-child relationships to children’s relationships with another important attachment figure—the teacher. Yet, a more limited number of studies have investigated the impact of teachers on children’s WM development, which contrasts with the extensive body of literature on parents. In line with the dynamic systems perspective, the literature points to associations between aspects of teacher-child interactions and classroom environments, on the one hand, and EF development, on the other hand (for a review see Vandenbroucke et al., 2018). By understanding the complex nature of these relationships, adults can work to create supportive environments that promote positive interactions and healthy development for children. Children interact with their teachers on the classroom level and on the dyadic level (i.e., teacher-student relationship—TSR) (Hamre and Pianta, 2007; Koomen et al., 2012).

Regarding TSR, several theories have been proposed to understand these relations (for a review see Spilt and Koomen, 2022). First, the self-determination theory, initially proposed by Deci and Ryan (1985), highlights three needs of the student in the classroom; namely, competence, autonomy, and relatedness. Classroom practices, as well as positive relationships with teachers, fostering feelings of competence, autonomy, and relatedness, are likely to result in student motivation required for learning and academic success (Ryan and Pintrich, 1997). Secondly, according to interpersonal theory (Horowitz and Strack, 2011), reciprocity (i.e., the importance of mutual responsiveness in relationships) promotes child development through positive feelings and emotional security (Kiesler, 1996; Locke and Sadler, 2007). In the school context, teachers, through their behaviour on a dyadic level (e.g., offering positive feedback and encouragement to the student), elicit behaviour from children (e.g., increased motivation or effort in their work), which, in turn, change teachers’ behaviour (e.g., investing more time and resources in supporting the student). Finally, attachment theory (Bowlby, 1969) proposes that the teacher acts as a “safe haven,” allowing the student to feel safe when exploring the classroom environment. In practice, students with a “safe haven” feel safe when making mistakes, and comfortable when faced with stress or (academic) challenges, both of which are necessary for continuous learning and improvement (Hedegaard, 2020). The latter theory is often the main framework applied in research on TSR; as a result, most commonly used measures to assess this relationship are grounded in the attachment theory [e.g., Student-Teacher Relationship Scale (STRS); Pianta, 2001]. Within this attachment perspective, the focus lies on the affective components of the relationship between a teacher and a specific student; more specifically, closeness, conflict, and dependency (Koomen et al., 2012; Verschueren and Koomen, 2012). Closeness refers to the degree of warmth, security, and open communication. Conflict refers to negative, unpredictable, and coercive teacher-student relationships. Dependency refers to the developmentally inappropriate degree of child’s reliance and possessiveness in the relationship.

Generally, research suggests positive associations between high-quality TSR and children’s WM development (Rimm-Kaufman and Hulleman, 2015; Course-Choi et al., 2017). Conversely, negative associations can be seen between low-quality TSR and children’s cognitive skills. For instance, conflicts with teachers can cause children to divert their attention from cognitive tasks toward emotional regulation. This diversion can strain WM resources, as children ruminate on the negative interactions rather than focussing on tasks at hand (Raver et al., 2012). Furthermore, conflictual relationships might lead to more behavioural problems, which can further detract from opportunities to engage in activities that support WM development (Blair and Raver, 2015). Importantly, the relationship between TSR (closeness and conflict) and child’s cognition and behaviour should be considered as bidirectional. Lower WM scores can be found to be related to increases in teacher-child conflict and decreases in teacher-child warmth while teacher-child conflict can be negatively associated with the development of WM (de Wilde et al., 2015). Important to consider that like most studies on TSR, the above mentioned reports only take teachers’ perception of the relationship into account while children’s perspectives on the quality of TSR are often neglected. Some studies, however, suggest that neither students nor teachers may evaluate their dyadic relationship objectively (Gilovich et al., 2000; Epley, 2008; Wu et al., 2010; Vervoort et al., 2014), suggesting that both teachers and students have their own unique perspectives on the TSR (Dong et al., 2021). Other studies revealed that each perception seems to predict different outcomes. More specifically, teacher’s perception better predicted teacher-rated outcomes while student’s perception—student-rated outcomes (Rey et al., 2007). Furthermore, while teachers’ perceptions of TSR predicted behavioural engagement, students’ perceptions predicted school belonging and arithmetic achievement (Hughes, 2011). Such findings highlight the unique, as well as overlapping, views each of the informants provide.

### Academic performance

In line with the dynamic systems perspective, research has established a clear relationship between WM and academic achievement. Overall, correlations between WM and academic achievement range from moderate to high (Nutley and Söderqvist, 2017). Most research on the association between cognitive abilities and academic performance treats cognitive skills as foundational constructs (i.e., primary abilities) leading to subsequent academic outcomes (i.e., secondary) (Sternberg et al., 2008). However, more recently, the unidirectional relation between cognitive abilities and academic performance has been challenged by the theory of mutualism, claiming that different skills and abilities become bidirectionally related during development as a consequence of mutually beneficial interactions between initially uncorrelated cognitive processes (van der Maas et al., 2006). Indeed, recent studies (Jacob and Parkinson, 2015; Follmer, 2017; Peng et al., 2018; Peng and Kievit, 2020) suggest that these constructs influence each other bidirectionally, as well as longitudinally (Schmitt et al., 2017; Miller-Cotto and Byrnes, 2020).

Similarly, studies on TSR at the dyadic level consistently indicate that a positive TSR and affective teacher behaviour are (longitudinally) associated with improved child engagement (Engels et al., 2021), motivation to learn (Pekrun et al., 2009), more profit from instruction (Crosnoe et al., 2010), improved cognitive processing (Ahnert et al., 2013), and academic performance (for meta-analyses see Roorda et al., 2011, 2017). Teachers who create a positive and supportive classroom environment and build relationships, characterised by warmth and care toward their students, can enhance students’ outcomes (Kiuru et al., 2015; Roorda et al., 2017; Pozo-Rico and Sandoval, 2020). Moreover, conflict-ridden relationships with teachers can lead to stress, anxiety and aggression in students (Zhou et al., 2021) and predict worse grades, work habits, and discipline problems (Longobardi et al., 2016; Özgan, 2016). Furthermore, the relationship between TSR and children’s academic performance may be bidirectional. Previous research suggests that teachers report more challenges (including less closeness and more conflict) in their relationships with students who have learning difficulties and disabilities compared to the students without (Zee et al., 2020). These challenges might arise due to various reasons, including student’s academic struggles, behavioural issues, or the teacher’s lack of resources or training to effectively support these students (Koenen et al., 2021).

### Current study

Taken together, there are indications for the bidirectional nature of the association between TSR and children’s WM, however, current knowledge on how these constructs affect each other over time (i.e., temporal relationship) is limited. More exploratively, this study aims to examine how WM and TSR relate to children’s academic performance.

The current study uses a longitudinal cross-lagged design (three time points throughout one school year) to explore the relationship between TSR: closeness and conflict (reported by both teacher and the child) and child’s WM: perceived WM problems (reported by both teacher and the parent) and performance WM (completed by the child) across time (Kearney, 2017). Three measurement points allow for insights into the bidirectional and temporal nature of the relationships between TSR and WM. Furthermore, this study aims to investigate bidirectional relationships between child’s academic performance: reading and arithmetic (completed by the child and assessed at the start of the year and at 5-month follow-up) and child’s WM, and TSR. In the current study, bidirectional relationships refer to relationships that have influences in both directions, meaning each construct affects the other over time (Allmann et al., 2021). Temporal relationships refer to unidirectional relationships that occur between different time points; they identify a sequence of influence from one construct to another over separate occasions (Last, 2007).

Based on the research findings presented above and taking into account the gaps in the current literature, two hypotheses are derived:

*Main hypothesis (1)*: There will be a bidirectional relationship between TSR and WM—better quality TSR (more closeness, less conflict) will be associated with better WM performance and fewer WM-related problematic behaviour in the classroom.

*Exploratory hypothesis (2)*: There will be bidirectional relationships between TSR (closeness and conflict) and academic performance (reading and arithmetic), and between WM (task performance and WM-related behaviour) and academic performance (reading and arithmetic).

## Materials and method

### Participants

The data was collected from children in the Flemish Region of Belgium (Flanders). A detailed overview of the socio-demographic participant data (child, parent, and teacher) collected at baseline can be found in the (under Supplementary materials, Appendices 1–3, respectively).

*Children*. Typically developing children (54 boys, 51.43%; 51 girls, 48.57%) between the ages of 6 to 12 years [*M*_*age*_ = 109.53 months (∼9.13 years), *SD* = 21.31 (∼1.78 years)], corresponding to grades 1 to 6 were included in the study. Children with an intellectual disability and children who take stimulant medication for improving EF functioning were excluded from the study. Based on the parent report, 8 children (7.62%) had physical difficulty, while 19 (18.10%)—had learning disability, with autism spectrum disorder (ASD) and attention deficit hyperactivity disorder (ADHD) being most prominent (*n* = 7, 6.67% and *n* = 3, 2.86%, respectively).

*Parents*. One parent/caregiver of the child (to account for diverse family structures) was invited to participate in the study. At baseline, 86 mothers (81.90%), 13 fathers (12.38%), and one other caregiver (0.95%) participated and completed the questionnaires. Based on the socio-demographic data provided by the parent, in the majority of cases (65.24%), at least one of the parents was highly educated—having obtained a bachelor’s or master’s degree [77 mothers (or a primary caregiver) (73.33%) and 60 fathers (or other caregiver) (57.14%)], and at least one of the parents was employed in a high-skilled or managerial position [53.81%: 58 mothers (or a primary caregiver) (55.24%) and 55 fathers (or other caregiver) (52.38%)], which is comparative to the Flemish average (Statbel, 2023).

*Teachers*. To be included in the study, teachers (*M*_*age*_ = 39.77 years, *SD* = 11.34) had to be from regular primary schools (i.e., special education schools were excluded), at least half-time teacher of the same class (in order to have established a stable relationship with the pupils), with at least 1 year of experience in education (in order to ensure that participating teachers have a baseline level of experience and confidence in their role) (*M* = 17.26 years, *SD* = 11.66).

### Procedure

The study was pre-registered through Open Science Framework (10.17605/OSF.IO/5CZG6) and ethical approval from the Social and Societal Ethics Committee at KU Leuven was obtained (G-2020-2355-R2). All deviations from the pre-registered protocol are described in more detail in (under Supplementary materials, Appendix 11).

Both parents and teachers, at three measurement points, received an online invitation to complete the questionnaires (described in detail below). Questionnaire completion took approximately 45 min at the first measurement point, 15—at the second measurement point, and 30 min at the third measurement point. Furthermore, virtual meetings were organised with participating children; these sessions took from half an hour to an hour, and involved filling in a questionnaire and completing WM and academic performance tasks (described in detail below). The overview of administered questionnaires and tasks is provided in (under Supplementary materials, Appendix 4). This study was part of a larger study (see the pre-registration for more details) and additional tasks and questionnaires were administered; however, only the ones relevant to the current study are presented here.

The recruitment started in September 2020, and data collection was completed in three waves. All three data collections had to take place during one school year considering that, in Belgian primary education, the child’s teacher changes every year (i.e., one teacher per year). Given that the study ran during the COVID-19 pandemic (i.e., school year of 2020–2021), data was collected while some restrictions were still in place (i.e., wearing face masks, social distancing). However, no data collection took place during the lockdown (regular primary schools were open, only the autumn and spring breaks were prolonged by an extra week). The first wave (i.e., baseline) was scheduled and completed in December 2020/January 2021 to allow teachers and students to develop and establish a dyadic relationship. Taking into account school holidays, the second wave (i.e., 3-month follow-up) took place in March/April 2021 [i.e., an average interval of 94 days (*SD* = 9.8)]. The final wave (i.e., 5-month follow-up) was realised in May/June 2021 to examine the relationship between teacher and student at the end of the school year, and their resultant WM and academic performance. The interval between wave 2 and 3, on average, was 68 days (*SD* = 14.4). Having three measurement points allowed for more perspective on possible changes and insight into the bidirectional and temporal nature of the relationships between TSR and WM, and the influence of TSR and WM on children’s academic performance later in the school year.

Participants were recruited by contacting school boards of primary schools in Flanders and asking to distribute the information letter to the teachers of these schools. Furthermore, an advertisement with a short description of the study and contact details of the key researchers was posted on the social media platforms and on Facebook groups for teachers and parents of primary school children. Once teachers agreed to participate, they received an information letter to be distributed to the parents of their students. The parents then let the researchers know if they and their child were interested in participating. If more than one student (within the same classroom) agreed to participate, only one of the students was randomly selected to form a teacher-student dyad. Informed consent from both teachers and parents was requested following an information sheet presented at the outset of the questionnaires. Participating children were asked to indicate whether they were willing to participate in the study (on a scale of three smileys: happy to participate, need more information, not willing to participate) before proceeding further with the questionnaires and tasks (i.e., informed assent). Pseudonymity was guaranteed by assigning a participant’s ID to the child and his/her parent and teacher for questionnaire completion and during the testing sessions with the children. The only document tying the participant’s name to their participant ID was the consent forms of the parents and teachers.

Children and their parents, as well as teachers, were provided with a chance to win a gift voucher worth 15 euros through the lottery (1/8 chance to win) as indicated in the information letter and informed consent form. The vouchers were given at the end of the data collection (i.e., 5-month follow-up). The data on the retention across time (per informant per wave) can be found in (under Supplementary materials, Appendix 5). In sum, 86.67% of the sample (or 91 participant triads) completed all the requested questionnaires and tasks.

### Measures

*Socio-demographic information*. Self-constructed questionnaires (based on an adaptation from Vandenbroucke et al., 2017) were administered to the teachers and parents (full information collected can be consulted through the pre-registration document and Supplementary materials, Appendices 2, 3). The questionnaire for the teacher assessed variables, such as gender, age, education level, and years of experience in education. A questionnaire administered to the parents assessed variables, such as family type, profession, education level of the parents, and parents’ age at first birth.

#### Teacher-student relationship: Closeness and Conflict

To measure the teacher-student relationship both measures from students and teachers were collected. Given that both perspectives were combined (see below for information on composite scores), only closeness and conflict domains could be used, as third domain appears to measure different aspects of TSR depending on the informant/questionnaire (i.e., dependency, autonomy, and negative expectations) (Spilt, 2010; Koomen and Jellesma, 2015).

A Dutch translation of the Student-Teacher Relationship Scale (STRS; Pianta, 2001; Koomen et al., 2012) was used to measure the teachers’ perception of the relationship. More specifically, three components of the relationship with the participating student: closeness, conflict, and dependency were assessed with a 28-item scale (e.g., “I share an affectionate, warm relationship with this child”, “This child and I always seem to be struggling with each other”, and “This child asks for my help when s/he really does not need help”, respectively). The five-point (1—definitely does not apply to 5—definitely applies) scale has excellent psychometric properties across multiple studies and samples (Pianta and Steinberg, 1992; Pianta et al., 1995; Koomen et al., 2007), including this study with internal consistency of 0.88 and 0.98 for conflict and closeness, respectively. Subscale scores were obtained by summing corresponding raw scores.

A translation of the Young Children’s Appraisals of Teacher Support (Y-CATS; Mantzicopoulos and Neuharth-Pritchett, 2003; Spilt et al., 2010) was used to measure the perceptions of the relationship between the teacher and the younger children (i.e., 6–8 year-olds). This questionnaire contains 27 items referring to three domains: warmth, conflict, and autonomy (e.g., “My teacher helps me when I do not understand”, “My teacher tells me that I am doing something wrong”, and “My teacher lets me do activities that I want to do”, respectively) that are rated by the child using a dichotomous response format—children are asked to indicate agreement (by placing a card in a safe) or disagreement (by placing the card in a trash can). Positive answers were scored 1 and negative answers—0. The Cronbach’s α values were 0.98 and 0.69 for warmth and conflict, respectively. The raw scores were summed for each subscale separately and divided by the total number of items within the subscale (i.e., each dimension obtains a score ranging from 0 to 1).

The Student Perception of Affective Relationship with Teacher Scale (SPARTS; Koomen and Jellesma, 2015) was administered to examine student-teacher relationship quality for the older age group (i.e., 9–12 year-olds). Children rated the extent to which they thought each of the 34 statements falling under closeness, conflict, and negative expectations domains (e.g., “My teacher understands me”, “Other children are punished less”, and “When I am with my teacher, I feel nervous”, respectively) applied to their relationship with the teacher on a five-point Likert scale (ranging from 1—“No, that is not true” to 5—“Yes, that is true”). The Cronbach’s α values were 0.74 and 0.55 for closeness and conflict, respectively, indicating moderate and low internal consistency (De Vellis, 2003). Mean subscale scores were calculated by summing the available scores of each subscale and dividing by the number of answers provided.

#### Working memory

WM was assessed using a multi-method, multi-informant approach, which is deemed necessary to have a reliable and sensitive measurement since WM is a multi-component and dynamic construct (Huizinga et al., 2018).

*Perceived WM Problems*. The Dutch version of the Behaviour Rating Inventory of Executive Function (BRIEF-2; Gioia et al., 2015; Huizinga and Smidts, 2020) was administered to both teachers and parents. A three-point Likert scale (1—never to 3—often) is used to record participant answers to 63 items falling under nine subscales. WM subscale contains 8 items and includes statements, such as “Forgets what to do when asked multiple things.” In this study, the Cronbach’s α values ranged from 0.79 to 0.86 across subscales, with 0.85 for WM subscale, for teacher reports while for parent reports, the values ranged from 0.77 to 0.86, with 0.86 for WM subscale. The raw scores were summed for each subscale separately. Correlation coefficient between teacher and parent report on the WM subscale, in the current study at baseline, was 0.38, *p* < 0.001—somewhat lower than those reported in the literature for the typically developing sample (ranging from 0.55 to 0.72) (Hendrickson and McCrimmon, 2019).

*Performance WM*. The Corsi block tapping test (Corsi, 1972) was used to assess child’s visuo-spatial WM. The test consists of nine blocks positioned randomly in front of the participant. An experimenter taps a subset of the blocks in a predetermined order while the participant observes. The participant is then asked to repeat the tapping order as presented (i.e., forward condition—tapping short-term auditory memory) or the order in reverse (i.e., backward condition—measuring the child’s ability to manipulate verbal information while in temporary storage). In the present study, a computerised version of the Corsi block tapping test was used. This approach allows for comparable span and error rates in comparison with the traditional version (e.g., Brunetti et al., 2014; Claessen et al., 2015). In this study, given short intervals between measurement points, instead of a span score, a total raw score of correct answers in the backward condition was used as an outcome variable. This approach was applied in order to capture small changes in children’s performance over time (e.g., a child with a span score of 5, could have a total raw score ranging from 5 to 8 points). The Cronbach’s α value indicated an acceptable consistency (0.72) for backward condition.

The Digit Span subtest (forward and backward conditions) of the Wechsler Intelligence Scale for Children—Fifth Edition (WISC-V; Wechsler, 2014) was administered to assess children’s verbal WM. The participating child was instructed to repeat a series of numbers (with increasing numbers of digits) forward and a different series of digits in reverse order. For consistency, the same approach as for the Corsi block tapping test (presented above) was applied when scoring the Digit Span subtest.

#### Academic performance

*Reading*. Children’s reading abilities were examined using the Een-Minuut-Test (EMT; Brus and Voeten, 1973)—measuring technical reading skills for existing words. In addition, Klepel-R (Klepel-Revised; van den Bos et al., 2019) was administered to the participating children. This test assesses the reading ability of pseudowords. The EMT and Klepel-R contain 116 words each and the outcome measures are the number of incoherent and pseudowords the child is able to read clearly within 1 or two 2 min (respectively) from a standard list of words. Only correctly read words (i.e., total read words minus misread words) were counted; these summed raw scores make up the outcome variables. Given that raw scores are more sensitive to small changes in performance, they can provide a more accurate reflection of the changes that occurred in the short periods of time. As raw scores, instead of norm scores, were used, participating children’s age at baseline (i.e., W1) was controlled for.

*Arithmetic*. Arithmetic of the participating children were assessed using the TempoTest Automatiseren (TTA; De Vos, 2010; suitable for first to sixth grade). This test consists of four parts: addition, subtraction, multiplication and division. Each part contains 50 items (equations) with increasing difficulty as the test progresses. The child is given 2 min for each of the parts; after the time is up, the child is asked to stop with the current sheet and continue to the next one. The outcome variable is the number of correctly completed equations within eight (two per part) minutes (allowing for corrections made during the testing), skipped equations or those completed incorrectly were, thus, not counted. Here too, participating children’s age at baseline was controlled for.

### Statistical approach

Missing data were handled using full information maximum likelihood estimation (FIML), allowing to maximise the data present, resulting in complete data for all participants (Allison, 2003).

Given a recent critique on treating WM and related abilities as latent variables (see Camerota et al., 2020), a composite variable approach was used to generate WM, TSR, and academic performance variables (in line with recent recommendations by Camerota et al., 2020; Howard et al., 2021). See Supplementary material for correlations between the variables (Appendices 7–9). As scores on each of the collected instruments varied significantly (regarding the scale used and the outcome variable obtained), the approach of summing standardised variables was used (see DiStefano et al., 2009). Composite scores were created by summing and averaging the z-scores of the outcome variables in each domain. The composite scores included:

1. “Perceived WM problems”: created by summing and averaging out BRIEF-2 teacher and parent reports on WM subscale. This data was collected at three time points: W1, W2, and W3.
2. “Performance WM”: created by summing and averaging out scores on backward conditions of Corsi block tapping and Digit Span tasks. This data was collected at three time points: W1, W2, and W3.
3. “Closeness”: created by summing and averaging out teacher report and either younger child or older child report (depending on the child’s age at the time of the measurement) on closeness subscale. This data was collected at three time points: W1, W2, and W3.
4. “Conflict”: created by summing and averaging out teacher report and either younger child or older child report (depending on child’s age at the time of the measurement) on conflict subscale. This data was collected at three time points: W1, W2, and W3.
5. “Arithmetic”: created by summing and averaging out four subtests of TempoTest Automatiseren: addition, subtraction, multiplication, and division. This data was collected at two time points: W1 and W3.
6. “Reading”: created by summing and averaging out two reading tasks: EMT and Klepel-R. This data was collected at two time points: W1 and W3.

Statistical software JASP (JASP Team, 2023, Version 0.17.1.0) was used to analyse the data. To test our theoretical (main and exploratory) models, cross-lagged structural equation modelling (SEM) was used. SEM is a powerful statistical technique that allows to examine relationships between multiple variables simultaneously (Kline, 2011). Our main model consisted of four composite variables measured at three time points: “Perceived WM problems,” “Performance WM,” “Closeness,” and “Conflict.” The model included regressions of four composite variables at Wave 2 (3-month follow-up) on their respective corresponding variables at Wave 1 (baseline). Additionally, variables at Wave 3 (5-month follow-up) were regressed on their corresponding variables at Wave 2. The exploratory model expanded upon the previous model by incorporating two additional variables related to academic performance: reading and arithmetic. The regressions in the exploratory model included all composite variables at Wave 3 regressed on their respective corresponding variables at Wave 1. All regressions were simultaneous, capturing the potential associations between the variables. To account for residual covariances, covariances between the error terms of the same constructs across waves were specified. Given that raw scores (and not norm) scores were used, in both models, participating children’s age at baseline (i.e., W1) was controlled for. The model fit was assessed using several fit indices, including root mean square error of approximation (RMSEA), comparative fit index (CFI), and Tucker-Lewis index (TLI). The RMSEA should be less than 0.05 for a good fit (Steiger, 1990), or between 0.05 and 0.08 for an acceptable fit (Browne and Cudeck, 1992), the CFI should exceed 0.90 for an acceptable fit, and 0.95 for a good fit to the data (Byrne, 1994), while TLI values exceeding 0.90 or over 0.95 indicate a good model fit (Hu and Bentler, 1999).

## Results

### Variable descriptives

The sample consisted of 105 children, their parents and their teachers. The mean values (z-scores) of composite scores at Wave 1 (baseline), Wave 2 (3-month follow-up), and Wave 3 (5-month follow-up) assessments are presented in (under Supplementary materials, Appendix 10).

### Correlation analysis

After missing data imputation, correlation analysis was conducted to explore the relationships between aggregated constructs at baseline, 3-month follow-up, and 5-month follow-up while controlling for participating child’s age (for the overview, see Table 1).

**TABLE 1:** Correlations between composite variables at baseline, at 3-month follow-up, and at 5-month follow-up (controlling for age).

Wave 1	1	2	3	4	5	6
1. Perceived WM problems	–					
2. Performance WM	−0.390***	–				
3. Closeness	−0.295**	0.121	–			
4. Conflict	0.364***	−0.07	−0.425***	–		
5. Arithmetic	−0.389***	0.273**	0.125	−0.112	–	
6. Reading	−0.347***	0.133	0.08	0.002	0.485***	–
**Wave 2**	**1**	**2**	**3**	**4**		
1. Perceived WM problems	–					
2. Performance WM	−0.243*	–				
3. Closeness	−0.275**	0.072	–			
4. Conflict	0.463***	−0.085	−0.591***	–		
**Wave 3**	**1**	**2**	**3**	**4**	**5**	**6**
1. Perceived WM problems	–					
2. Performance WM	−0.215*	–				
3. Closeness	−0.309**	−0.012	–			
4. Conflict	0.421***	−0.048	−0.516***	–		
5. Arithmetic	−0.418***	0.269**	0.218**	−0.143	–	
6. Reading	−0.256**	0.076	0.109	−0.089	0.540***	–

Conditioned on variables: participant age (in months).

***p < 0.001, **p < 0.01, *p < 0.05.

At baseline, the results showed significant negative correlations between Perceived WM problems and Performance WM, Closeness, Arithmetic, and Reading, while a significant positive correlation was found between Perceived WM problems and Conflict. Furthermore, a significant positive correlation was found between Performance WM and Arithmetic, and between Arithmetic and Reading. Not surprisingly, a significant negative correlation was found between Closeness and Conflict.

At the 3-month follow-up, the results followed a similar pattern. Perceived WM problems negatively correlated with Performance WM and Closeness, while positively correlated with Conflict. Furthermore, a significant negative correlation was found between Closeness and Conflict.

At 5-month follow-up, the patterns remained. Perceived WM problems negatively correlated with Performance WM, Closeness, Arithmetic, and Reading, but positively with Conflict. Performance WM and Arithmetic, as well as Arithmetic and Reading, were positively correlated, while Closeness and Conflict—negatively. A significant positive correlation was found between Closeness and Arithmetic—such correlation was only evident at 5-month follow-up. These findings suggest that associations described above are stable across time as evident by the almost identical results throughout the three waves.

### Structural equation modelling (SEM) analysis

**FIGURE 1:**
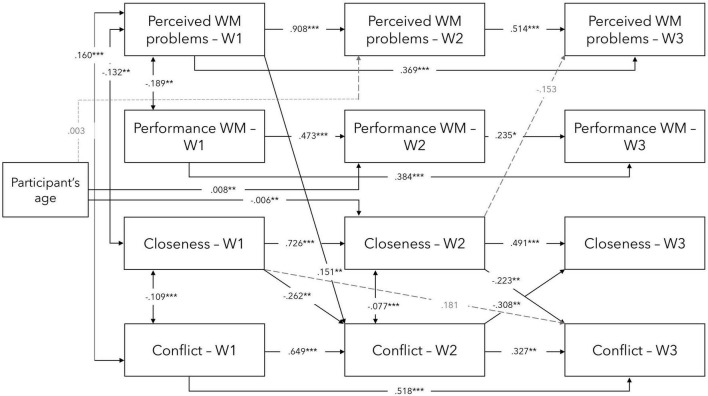
Visual representation of significant and near-significant direct effects in the main model 1 between TSR and WM variables at waves 1, 2, and 3. W1—Wave 1/Baseline, W2—Wave 2/3-month follow-up, W3—Wave 3/5-month follow-up. Controlled for participants’ age at baseline. ***p < 0.001, **p < 0.01, *p < 0.05; grey, dashed lines indicate near-significance.

Firstly, the bidirectional and temporal relationship between TSR and WM was explored. The first model (Model 1 or the full model; see Figure 1) included auto-regressive and cross-lagged direct paths between TSR (Closeness and Conflict) and WM (Perceived WM problems and Performance WM) at three time points (baseline, 3-month follow-up, and 5-month follow-up) while controlling for participants’ age at baseline. The results showed that Model 1 had a good fit with the data, as evidenced by the AIC and BIC values (1830.148 and 2086.430, respectively), the CFI of 0.957 and TLI of 0.942, while the RMSEA was 0.062. The values presented are unstandardised beta coefficients.

**FIGURE 2:**
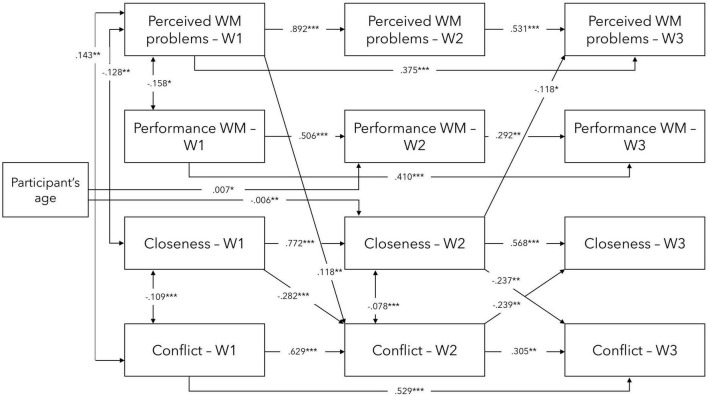
Significant direct effects in the main model 2 between TSR and WM variables at waves 1–3. W1—Wave 1/Baseline, W2—Wave 2/3-month follow-up, W3—Wave 3/5-month follow-up. Controlled for participants’ age at baseline. ***p < 0.001, **p < 0.01, *p < 0.05.

By running Model 1, with all the pathways included, the relationships between all of the variables in the model were examined. This is a useful starting point to gain an understanding of the overall relationships among the variables. However, by exploring the regression coefficients in Model 1 and identifying and including only the significant predictors, a more parsimonious model can be created (based on Bentler and Mooijaart, 1989). In order to streamline the model and focus on the most meaningful relationships, only significant and near-significant (i.e., 0.05 < *p* < 0.10) effects from Model 1 in Model 2 were retained (see Figure 2). Significant effects indicate robust associations between variables, suggesting the presence of a meaningful relationship. Near-significant effects, although not reaching conventional levels of statistical significance, may still suggest a trend or potential relationship that warrants further investigation. Model 2 had AIC and BIC values of 1778.104 and 1921.935, respectively, and the CFI of 0.955 and TLI of 0.926. The RMSEA was 0.064 and the 90% CI for RMSEA ranged from 0.040 to 0.088.

Based on the various model fit indices, both models appear to provide a good fit for the data, with slight improvement seen in Model 2 (given lower AIC and BIC values). However, the second model (Model 2) is more parsimonious as it includes only significant and near-significant predictors identified in Model 1. Therefore, Model 2 seems to provide a simpler and more straightforward explanation of the relationships between the variables. However, there is no proof for a significant improvement over Model 1 [Δχ^2^ (33) = 27.61, *p* = 0.732].

Overall, each of the variables significantly predicted their consecutive measurement outcome, and Wave 1 (baseline) scores predicted outcomes at Wave 3 (5-month follow-up) for Perceived WM problems, Performance WM, and Conflict, but not Closeness. Furthermore, significant cross-lagged effects were found. More specifically, Perceived WM problems at baseline predicted Conflict at 3-month follow-up while Closeness at 3-month follow-up predicted Perceived WM problems at 5-month follow-up. In addition, Closeness at baseline predicted Conflict at 3-month follow-up, which, in turn, predicted Closeness at 5-month follow-up, and Closeness at 3-month follow-up predicted Conflict at 5-month follow-up.

**FIGURE 3:**
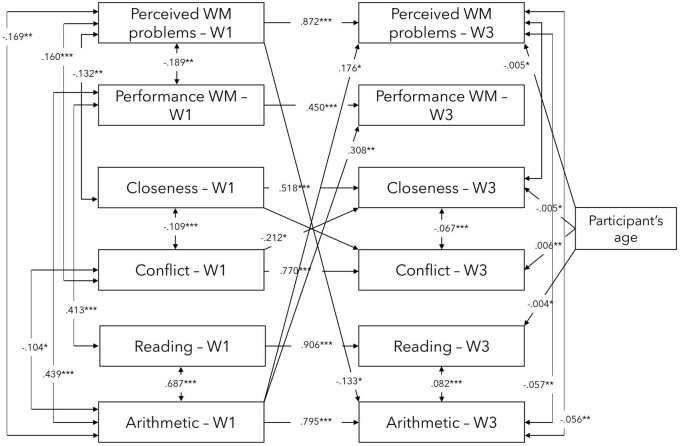
Visual representation of significant direct effects in the exploratory model 1 between WM, TSR, and academic performance variables at waves 1 and 3. W1—Wave 1/Baseline, W3—Wave 3/5-month follow-up. Controlled for participants’ age at baseline. ***p < 0.001, **p < 0.01, *p < 0.05.

Secondly, the exploratory hypothesis assuming there will be bidirectional relationships between TSR and academic performance, as well as between WM and academic performance, was tested. The first model (Model 1; see Figure 3) included direct paths between TSR (Closeness and Conflict), WM (Perceived WM problems and Performance WM), and academic performance (Arithmetic and Reading) at two time points (baseline and 5-month follow-up), while controlling for participants’ age at baseline. Model 1 had an AIC of 2006.251 and a BIC value of 2257.303, and the CFI of 0.961 and TLI of 0.945. While the RMSEA value was 0.073, the 90% CI for RMSEA fell between 0.0045 and 0.103.

**FIGURE 4:**
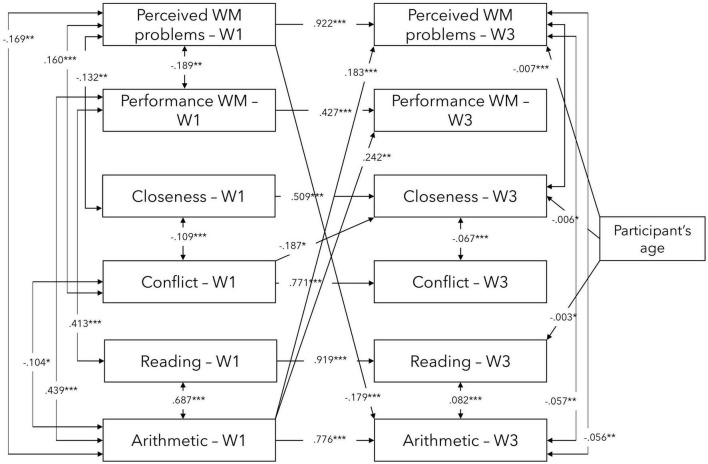
Significant direct effects in the exploratory model 2 between WM, TSR, and academic performance variables at waves 1 and 3. W1—Wave 1/Baseline, W3—Wave 3/5-month follow-up. Controlled for participants’ age at baseline. ***p < 0.001, **p < 0.01, *p < 0.05.

Furthermore, the regression coefficients in Model 1 were explored to identify significant predictions (the values presented are unstandardised beta coefficients). Only the significant predictions were retained in Model 2 (see Figure 4). The model had AIC value of 2075.432 and BIC value of 2232.015, the CFI of 0.964 and TLI of 0.934. The RMSEA value was 0.056, while the 90% CI for RMSEA between 0.000 and 0.100. However, there may be some unexplained variation in the model that could be further explored.

Overall, each of the variables at baseline significantly and strongly predicted their 5-month follow-up measurement outcome. Furthermore, significant cross-lagged effects between variables were found. More specifically, Perceived WM problems at baseline predicted Arithmetic at Wave 3 (5-month follow-up) while Arithmetic at baseline, in turn, predicted Perceived WM problems and Performance WM at 5-month follow-up. Finally, Conflict at baseline predicted Closeness at 5-month follow-up.

## Discussion

The present longitudinal study aimed to examine the bidirectional relationships between WM, TSR, and academic performance in primary school, and temporal relationship between WM and TSR, and made further contributions to the existing literature.

First, somewhat in line with previous correlational studies (e.g., de Wilde et al., 2015; Devine et al., 2016; Sosic-Vasic et al., 2017), an association between TSR and WM was found. The results within each of the waves indicate that better quality TSR (i.e., closer and less conflictual relationship) was associated with fewer WM-related problematic behaviour in the classroom, but not correlated with children’s performance on WM tasks.

No bidirectional relationship was found between TSR (neither closeness nor conflict) and WM (neither perceived WM problems nor performance WM). Therefore, our main hypothesis was unsupported. The current study, however, has found a temporal relationship over time between TSR and WM-related problem behaviour. In particular, more WM-related problematic behaviour in the classroom at baseline were associated with poorer TSR quality (characterised by more conflict) at 3-month follow-up (small effect <0.20), while better TSR quality (characterised by more closeness) at 3-month follow-up was associated with fewer WM-related problematic behaviour at 5-month follow-up (small effect <−0.20). This result suggests that, on the one hand, the quality of TSR plays an important role in students’ cognitive functioning and classroom behaviour, while children’s cognitive abilities can, on the other hand, affect children’s relationships with their teacher. Such findings could be explained by the idea that children with less WM-related challenges tend to be more engaged in activities in the classroom and display fewer disruptive WM-related problems (e.g., Hughes and Kwok, 2007; Colmar and Double, 2017). Such positive behaviour might meet the expectations of the teacher and evoke more positive reactions and interactions with the student (Jing et al., 2020; Rudasill et al., 2020; Sun, 2021; Haldimann et al., 2023). As a result, positive interactions can then lead to teachers providing more support and attention to the child (Klem and Connell, 2004; Hamre and Pianta, 2005). Previous research shows that teachers tend to provide more scaffolding and verbal instruction for children with whom they seem to have a closer relationship (Hughes and Ensor, 2009; Nomi, 2009), which, as a result, can promote WM abilities. This finding suggests that positive TSR may act as a protective factor against WM problems in children, highlighting the importance of interventions focussing on improving or facilitating positive relationships between teacher and student (Bosman et al., 2021). However, it is also important to take into consideration method variance. Generally, variables measured using the same method (e.g., two different questionnaires on the same construct) tend to show stronger correlations with each other than variables measured through different methods (e.g., a neuropsychological test and a questionnaire on the same construct; Podsakoff et al., 2003). Furthermore, the teacher reported on both TSR quality and children’s WM-related problems, which might have led to some bias (e.g., teachers who have a close relationship with the child, might be more favourable in rating child’s behaviour). However, the latter effect is minimised by including other informants’ reports (child and parent) in composite variables. On the other hand, TSR might be hindered by already existing WM-related, as well as behavioural problems exhibited by the child. These difficulties could result in somewhat negative interactions with teachers, potentially leading to strained TSR. TSR might be difficult to improve if such issues persist, thus early interventions aimed at improving children’s WM skills and managing problematic behaviour in the classroom could potentially alleviate some of these challenges. By addressing these difficulties early on, student’s academic performance and behaviour might improve, which could help build a more positive TSR (Diamond and Lee, 2011; Spilt et al., 2012).

Surprisingly, children’s performance on WM tasks was not related to TSR (and vice versa). Given that WM-related problematic behaviour was reported by the teachers and parents, this variable is considered as perceived WM. This perceived WM might be more important for a relationship between teacher and student rather than the “actual WM” performance. “Actual WM” performance is measured by tasks conducted in a relatively controlled, structured, stimulus-free environment and might not reflect children’s WM abilities and difficulties experienced in a daily life environment, such as the classroom (for a review, see Souissi et al., 2022). It is widely known that performance on tasks and behaviour ratings do not correlate well, therefore, confirming the notion that these two assessment types seem to measure two different aspects of the same construct or these performances are highly context-dependent (Miyake et al., 2000; Hughes and Graham, 2002). This idea is also supported by the current study as children with more perceived WM problems tended to have poorer performance on WM tasks, however, only evident at baseline, while no correlation was found at later points in time.

No associations between TSR and academic performance at baseline were found in this study, while, at the 5-month follow-up, only one correlation—between children’s arithmetic performance and closeness was revealed. Such findings are inconsistent with previous research that found positive associations between quality of TSR and academic achievement in children (Hamre and Pianta, 2005; Hughes and Coplan, 2010; Roorda et al., 2011), and literature highlighting the importance of a supportive and positive learning environment for academic success (Roorda et al., 2011; Pekrun et al., 2017).

Furthermore, no bidirectional relationship was found between TSR (neither closeness nor conflict) and children’s academic performance neither on reading nor arithmetic. Such results might be explained by the same approach discussed regarding WM assessment. Academic performance tasks, used in this study, were highly structured and very brief, and were administered in the conditions of stress-free home environment. However, school work (for instance, examining children’s assignments, classwork, grades obtained in real academic settings) could provide valuable insights and capture the child’s performance in a real-life context (i.e., the academic environment). Indeed, sometimes, researchers opt for standardised academic performance tests that are administered by the schools across the country rather than tests administered solely for the purpose of research. These standardised achievement tests are designed to measure students’ academic knowledge and skills in a systematic way across classrooms, and might provide a more representative and comparable measure (Paris and Paris, 2001). To our knowledge, there is no universal assessment used across studies on academic performance as these highly depend on the context, age range, and country where the study took place, therefore, highlighting the need of a standardised assessment measure. Not found association between TSR and academic performance can also be seen in a positive light—suggesting that potentially children’s academic performance might not strongly affect teacher’s relationship with the children. Other aspects, such as child’s engagement, motivation, and participation in the classroom activities might play a more significant role (Martin and Marsh, 2006; Timoštšuk and Näkk, 2019). In further research, these additional variables should, therefore, be taken into consideration as well.

Moreover, the current study found associations between WM and children’s academic performance (at W1 and W3). More specifically, children exhibiting more WM-related problematic behaviour tended to have poorer academic performance (as indicated by both arithmetic and reading tasks), while children with better performance on WM tasks tended to perform better at arithmetic (but not reading, both at W1 and W3). These findings are consistent with previous research that found associations between WM abilities and academic achievement in children (Gathercole and Alloway, 2008; Willoughby et al., 2019; Peng and Kievit, 2020).

More importantly, the exploratory hypothesis was partially supported by this study. The findings showed that arithmetic performance and perceived WM problems were bidirectionally related. More specifically, children’s arithmetic performance at baseline was positively related to fewer WM-related problematic behaviour in the classroom at 5-month follow-up (small effect <0.20). While children’s WM-related problematic behaviour at baseline were related to children’s arithmetic performance at 5-month follow-up (small effect <−0.20). Furthermore, a positive association was found between children’s arithmetic performance at baseline and children’s performance on WM tasks (medium effect <0.30). These results are consistent with previous research that has demonstrated the importance of WM in academic outcomes as a predictor of academic achievement in children (Gathercole et al., 2008; Alloway and Alloway, 2010; Cortés Pascual et al., 2019; Willoughby et al., 2019; Peng and Kievit, 2020). These findings also suggest that students with stronger WM abilities are better equipped to manage the demands of academic tasks and, as a result, are more likely to perform better on academic assessments. However, given the bidirectional relationship, alternatively, children with better arithmetic skills might receive more opportunities to develop and improve their WM by the teacher, which, in turn, facilitates subsequent learning behaviour and further academic development (Mattera et al., 2021; ten Braak et al., 2021). Important to note that such associations were not found between WM and reading performance. The findings may vary based on the specific tasks and cognitive processes involved. Most arithmetic tasks rely heavily on WM, as solving an arithmetic problem requires holding a set of numbers in mind, manipulating this information, and remembering the intermediate results (Fuchs et al., 2012). Reading, while certainly involving WM, also heavily relies on other skills, such as vocabulary knowledge, not taken into account in our measurement. Furthermore, WM performance tasks, administered in this study, were also related to the arithmetic domain, including spatial and number recall, which might be part of the explanation of the stronger associations with this domain.

### Limitations and future directions

A limitation of the current study is that the sample mainly included typically developing children, primarily from high-SES backgrounds. Future studies should include a more diverse sample and investigate whether these findings generalise to other populations, such as children from low-SES backgrounds or those with disabilities. Furthermore, given that the recruitment took place during the COVID-19 pandemic, the recruitment of the teachers proved to be quite challenging as seen by a decreased interest in participation (as schools and teachers were already overwhelmed adjusting to the new requirements, staff and student absences and changes in the curriculum), which might have led to a recruitment of somewhat biased sample—comprised of those most motivated. During the COVID-19 pandemic, several regulations were in place in the classrooms, such as wearing of the masks, distancing and avoiding physical contact between the teacher and the children. Regular primary schools were open (no school closures), only the autumn and spring breaks were prolonged by an extra week. Verbal expressions, smiles and supportive physical touch (i.e., hand on shoulder, hugs) play an important role in TSR, especially with younger children, which were somewhat reduced. Teacher and child absences might also have played a role in affecting these interactions. Even though, these restrictions might have contributed to the variance in the present findings, the full impact of COVID-19 pandemic and implemented measures is not fully understood. Initially, observations of interactions between the teacher and participating child were planned to take place in the classrooms. However, to avoid the risks posed to the researchers, children and school staff, no visits took place. Finally, the testing sessions took place online, which made it difficult to always ensure a controlled, stimulus-free environment when completing academic performance tasks. Even though there are indications that computerised version of the Corsi block-tapping task provides comparable results with the traditional version (e.g., Brunetti et al., 2014; Claessen et al., 2015), these results were found in the adult population. The computerised version might be somewhat more difficult to complete, especially by young children, who are not a familiar with using a computer mouse, causing a delay and, therefore, poorer recall of the items.

The current study, nevertheless, has several strengths. Firstly, a longitudinal cross-lagged design allows for new insights into changes of these constructs over time (i.e., over one school year), as well as into bidirectional and temporal relationships between these constructs, which remains somewhat understudied. Secondly, current study applied a multi-method, multi-informant approach to assess a complex construct of WM, which has revealed significant differences between WM performance tasks and perceived WM highlighting the need for using diverse measurement or interpreting results with caution (i.e., context-specific). Finally, the TSR was rated not only by the teacher (as most commonly assessed), but also from a child’s perspective, providing a more nuanced understanding of TSR. Combining both perspectives assumes that both are equally valid and the combination, therefore, represents a more “objective” evaluation of the relationship. However, teachers and students may have different interpretations of the same events and behaviours, subjected to bias. Children might be more sensitive to the negative interactions with their teacher, as well as affected by peer influence, thus underestimating the TSR quality, while teachers might want to appear more caring and competent (i.e., social desirability bias) and overestimate the positive aspects of the relationship (Sandilos et al., 2016; Roorda et al., 2017). Nevertheless, both reports provide valuable insights and hint toward the complexity of such relationships. It is important to consider that, in the current study, the teacher and child reports did not correlate significantly, introducing complexities in combining the perspectives into one measure. However, the lack of correlation can be perceived not as a limitation but rather as an illustration of the richness and depth of the relationship, in line with the dynamic systems approach (Spilt et al., 2012). Such findings suggest that an observation by a teacher (and, in addition, a third person, such as a researcher) could provide not only an independent measure of this relationship, but a valuable insight into specific interactions in the classroom and displayed WM-related problematic behaviour by the child; and vice versa for the child’s perspective. This point should be taken into account in future research on TSR and interactions on the classroom level. While the current study highlighted the links between children’s WM, their academic performance, and TSR quality, it is important to highlight the broader context of such findings. Understanding this multifaceted relationship better could pave the way for more targetted teaching strategies, benefitting from the strengths of students’ EF. Moreover, the quality of TSR could play a pivotal role in mediating the effects between cognitive abilities and academic outcomes. Future research should explore these nuances, broadening the scope beyond WM. By delving deeper into how EF as a whole influence learning trajectories and how they interplay with classroom dynamics, should provide a more comprehensive understanding, informing specific training programmes and interventions, ultimately enhancing educational outcomes for students.

## Conclusion

The present study contributes to the existing literature by providing valuable insights into the associations and bidirectional and temporal relationships between WM, TSR, and academic performance in primary school children. The applied design allowed to assess how these constructs affect each other across time and provide more insight into the nature of these relationships. Overall, the findings of this study provide evidence of the complex interplay between WM, TSR, and academic performance. The findings are consistent with previous research that has shown the importance of the TSR in WM performance (Roorda et al., 2011; Pekrun et al., 2017) and the role of WM in academic performance (Gathercole et al., 2008; Alloway and Alloway, 2010). These results suggest that relationships between the teacher and students play an important role in supporting students’ cognitive and academic development. The findings suggest that positive TSR may serve as a protective factor against WM problems and that interventions that target both TSR and WM may have positive effects on children’s academic performance. Furthermore, the findings of this study emphasise the importance of identifying and addressing WM-related problematic behaviour early in a child’s academic career to promote positive academic outcomes. These findings have important implications for practice and highlight the need for interventions that promote positive TSR and, in turn, improve cognitive and academic outcomes in children.

## Data availability statement

The data that support the findings of this study are available upon reasonable request.

## Ethics statement

The studies involving humans were approved by the Social and Societal Ethics Committee at KU Leuven. The studies were conducted in accordance with the local legislation and institutional requirements. Written informed consent for participation in this study was provided by the participants (teachers and parents) and participating children’s legal guardians/next of kin.

## Author contributions

SS, MH, and DB designed the study and developed research questions. PW provided valuable insights at the early stages of the study. JD involved in the planning and implementation of the data collection. SS supervised the recruitment process and data collection. SS analysed and interpreted the data, and completed the writing of the manuscript, with valuable contributions from MH, PW, and DB. MH, PW, and DB provided guidance, and critical and constructive feedback. DB supervises the project and provided continuous support and critical revision at every step of the study process and article writing. All authors contributed to the conception of the research idea, contributed to the article and approved the submitted version.
