# Supplementary material for: The association between working memory, teacher-student relationship, and academic performance in primary school children
Source: Front Psychol. 2023 Sep 22;14:1240741. doi: 10.3389/fpsyg.2023.1240741 (PMC10556679; doi:10.3389/fpsyg.2023.1240741)
Supplement: Supplementary file 1 [file Data_Sheet_1.pdf]

Appendix 1. *Child Demographic Information.*

Characteristic	*n*	%
Gender (*n* = 105)		
Male	54	51.43
Female	51	48.57
Age in months (*n* = 101)		
73-84	16	15.24
85-96	17	16.19
97-108	14	13.33
109-120	15	14.29
121-132	23	21.90
133-144	16	15.24
Physical difficulties (*n* = 102)		
No	94	89.52
Yes	8	7.62
Mental difficulties (*n* = 100)		
No	81	77.14
Yes	19	18.10
ADHD	3	2.86
ASD	7	6.67
Anxiety disorder	1	0.95
Trauma-related disorder	1	0.95
Other	9	8.57
Premature birth (*n* = 103)		
No	94	89.52
Yes	9	8.57
Repeated year (*n* = 103)		
No	102	97.14
Yes	1	0.95

Difficulties in school (*n* = 103)		
No	84	80.00
Yes	19	18.10
French	4	3.81
Learning	5	4.76
Physical education	3	2.86
Musical education	1	0.95
Dutch	9	8.57
Social skills	1	0.95
Mathematics	9	8.57
Other	4	3.81
Receiving support (*n* = 103)		
No	4	3.81
Yes	15	14.29
Extra support in class	11	10.48
Extra exercises at home	5	4.76
Extra after-school guidance	5	4.76
Other	2	1.90

Appendix 2. *Parent Demographic Information.*

Characteristic	*n*	%
Relation to the child (*n* = 100)		
Mother	86	81.90
Father	13	12.38
Caregiver	1	0.95
Family structure (*n* = 103)		
Two-parent family	75	71.43
One-parent family	10	9.52
Other	18	17.14
Number of children (*n* = 103)		
One	13	12.38
Two	53	50.48
Three	27	25.71
Four	9	8.57
Five	1	0.95
Number of people living in the house (*n* = 103)		
One	2	1.90
Two	5	4.76
Three	10	9.52
Four	53	50.48
Five	24	22.86
Six	8	7.62
Seven	1	0.95
Number of rooms in the house (*n* = 103)		
3	3	2.86
4	5	4.76
5	13	12.38
6	21	20.00
7	25	23.81
8	14	13.33
9	10	9.52
10	6	5.71
11	3	2.86
12	2	1.90
13	1	0.95

Age of the mother at first birth (n = 103)		
25 or under	20	19.05
26-30	54	51.43
31-35	20	19.05
36 or above	8	7.62
Age of the father at first birth (n = 101)		
25 or under	6	5.71
26-30	40	38.10
31-35	32	30.48
36 or above	23	21.90
Age of the father at first birth (n = 101)		
25 or under	6	5.71
26-30	40	38.10
31-35	32	30.48
36 or above	23	21.90
Diploma of the mother (n = 103)		
Secondary education	25	23.81
Bachelor	41	39.05
Master	36	34.23
Other	1	0.95
Diploma of the father (n = 101)		
Secondary education (or less)	35	33.33
Bachelor	32	30.48
Master	28	26.67
Other	6	5.71
Profession of the mother (n = 99)		
Skilled worker	4	3.80
Farmer or self-employed worker	7	6.67
Lower-level employee	30	28.57
Higher-level employee	52	49.52
Management	6	5.71

Profession of the father (n = 100)		
Unskilled worker	7	6.67
Skilled worker	11	10.48
Farmer or self-employed worker	16	15.24
Lower-level employee	11	10.48
Higher-level employee	50	47.62
Management	5	4.76
Employment of the mother (n = 101)		
Full-time	76	72.38
Part-time	23	21.90
Voluntarily non-employed	2	1.90
Employment of the father (n = 100)		
Full-time	95	90.48
Part-time	6	5.71
Nationality of the mother (n = 102)		
Belgian	98	93.33
Other	4	3.81
Nationality of the father (n = 103)		
Belgian	97	92.38
Other	5	4.76
Nationality of the child (n = 103)		
Belgian	102	97.14
Other	1	0.95
Mother tongue of the mother (n = 103)		
Dutch	100	95.24
Other	3	2.86
Mother tongue of the father (n = 103)		
Dutch	98	93.33
Other	4	3.81
Mother tongue of the child (n = 103)		
Dutch	102	97.14
Other	1	0.95

Appendix 3. *Teacher Demographic Information.*

Characteristic	*n*	%
Gender (*n* = 104)		
Male	10	9.52
Female	94	89.52
Age in years (*n* = 102)		
25 or under	9	8.57
26-35	29	27.62
36-45	25	22.89
46-55	26	25.30
56 or over	13	10.84
Experience in years (*n* = 104)		
1-5	21	20.00
6-15	31	29.52
16-25	22	20.95
26-35	20	19.05
36 or more	10	9.52
Class size (*n* = 104)		
minimum	12	/
maximum	39	/
Mean	20.36	/
SD	4.36	/

Appendix 4. *Overview of the Administered Tasks and Questionnaires per Wave.*

Tasks / questionnaires	Parent			Teacher			Child		
	W1	W2	W3	W1	W2	W3	W1	W2	W3
Socio-demographic questions	x			x					
STRS				x	x	x			
BRIEF-2	x	x	x	x	x	x			
Y-CATS / SPARTS							x	x	x
Corsi block tapping task							x	x	x
Digit Span subtest							x	x	x
Arithmetic tasks							x		x
Reading tasks							x		x

Note: W1 – Wave 1/Baseline, W2 – Wave 2/3-month follow-up, W3 – Wave 3/5-month follow-up, STRS – Student-Teacher Relationship Scale, BRIEF-2 – Behaviour Rating Inventory of Executive Function, Y-CATS – Young Children's Appraisals of Teachers Support, SPARTS – Student Perception of Affective Relationship with Teacher Scale.

Appendix 5. *Overview of the Number of Participants Completing Tasks and Questionnaires (per Informant, per Wave)*.

Tasks / questionnaires	W1	W2	W3
Parent questionnaires	103	104	101
Teacher questionnaires	104	103	101
Child questionnaire	105	103	102
Child WM tasks	104	103	100
Child academic performance tasks	102	/	99

Note: W1 – Wave 1/Baseline, W2 – Wave 2/3-month follow-up, W3 – Wave 3/5-month follow-up.

Appendix 6. *Mean Scores (before the imputation of missing data) of the Sample at Baseline and Follow-UpAssessments.*

Variables	W1			W2			W3		
	n	M	SD	n	M	SD	n	M	SD
Perceived WM problems – teacher	103	11.61	3.34	102	11.86	3.83	101	11.30	3.50
Perceived WM problems – parent	100	12.80	3.68	102	12.66	3.06	101	12.90	3.54
Performance WM – Corsi	105	6.46	1.95	100	6.65	1.65	101	6.58	1.98
Performance WM – WISC	105	8.08	2.29	103	8.30	2.44	101	7.58	2.60
Closeness – teacher	103	42.97	6.02	98	41.99	6.48	101	42.65	6.49
Closeness – younger child*	49	0.88	0.11	44	0.92	0.09	42	0.90	0.18
Closeness – older child*	53	31.47	4.90	59	29.46	6.56	57	29.74	5.62
Conflict – teacher	103	16.44	6.65	98	17.33	6.35	101	16.66	6.15
Conflict – younger child*	49	0.26	1.88	44	0.20	0.18	42	0.16	0.18
Conflict – older child*	53	14.45	3.47	59	15.49	5.99	57	14.63	4.87
Arithmetic – addition	103	28.05	10.80				97	31.54	10.26
Arithmetic – subtraction	103	25.62	11.87				97	29.37	11.28
Arithmetic – multiplication	94	26.11	14.36				85	31.31	12.90
Arithmetic – division	93	26.45	16.19				84	32.50	14.12
Reading – Klepel-R	105	42.61	22.46				101	45.85	18.56
Reading – EMT	105	47.45	21.94				101	53.40	18.66

Note: W1 – Wave 1/Baseline, W2 – Wave 2/3-month follow-up, W3 – Wave 3/5-month follow-up.
*For this measure different questionnaires had to be used: Y-CATS for the younger children (*n* = 48) and SPARTS for the older children (*n* = 57).

Appendix 7. *Correlations Between Collected WM Variables (and age) at Baseline.*

Variable	1	2	3	4	5
1. Participant age (in months)	–				
2. Perceived WM – Teacher	0.01	–			
3. Perceived WM – Parent	0.07	0.38***	–		
4. Performance WM – Corsi	0.54***	-0.25*	0.05	–	
5. Performance WM – WISC	0.47***	-0.26**	-0.21*	0.33***	–

**p* < .05, ***p* < .01, ****p* < .001

Appendix 8. *Correlations Between Collected TSR Variables (and age) at Baseline.*

Variable	1	2	3	4	5	6	7
1. Participant age (in months)	–						
2. Closeness – Teacher	-0.14	–					
3. Conflict – Teacher	-0.17	-0.30**	–				
4. Closeness – Older child	0.11	0.19	-0.22	–			
5. Conflict – Older child	0.10	0.15	0.10	-0.17	–		
6. Closeness – Younger child	0.12	-0.06	0.06	/	/	–	
7. Conflict – Younger child	-0.11	-0.03	-0.07	/	/	-0.07	–

*$p < .05$, **$p < .01$, ***$p < .001$

Appendix 9. *Correlations Between Collected Academic Performance Variables (and age) at Baseline.*

Variable	1	2	3	4	5	6	7
1. Participant age (in months)	–						
2. Arithmetic – Addition	0.76***	–					
3. Arithmetic – Subtraction	0.72***	0.93***	–				
4. Arithmetic – Multiplication	0.64***	0.80***	0.84***	–			
5. Arithmetic – Division	0.62***	0.78***	0.80***	0.94***	–		
6. Reading – Fluency	0.74***	0.84***	0.80***	0.76***	0.74***	–	
7. Reading – Decoding	0.68***	0.72***	0.71***	0.59***	0.60***	0.87***	–

**p* < .05, ***p* < .01, ****p* < .001

Appendix 10. *Mean Values (z-scores) of Composite Variables at Baseline and Follow-Up Assessments.*

Variables	W1		W2		W3	
	M	SD	M	SD	M	SD
Perceived WM problems	3.347×10^{-17}	0.87	8.02×10^{-17}	0.86	1.88×10^{-16}	0.87
Performance WM	2.40×10^{-17}	0.80	5.22×10^{-17}	0.80	-1.23×10^{-17}	0.80
Closeness	0.41	0.53	0.40	0.62	0.38	0.62
Conflict	-0.40	0.53	-0.37	0.60	-0.43	0.58
Arithmetic	1.90×10^{-17}	0.91			7.02×10^{-17}	0.89
Reading	4.02×10^{-18}	0.97			-1.05×10^{-16}	0.94

Note: W1 – Wave 1/Baseline, W2 – Wave 2/3-month follow-up, W3 – Wave 3/5-month follow-up; M – mean, SD – standarddeviation, WM – working memory.

Appendix 11. *Deviations from the Pre-registered Protocol.*

1. Regarding the sample, no clinical group was recruited. Unfortunately, efforts to recruit a clinical sample (more specifically, autistic children) did not result in enough participants to create two comparable groups. Instead, autistic children who attended regular education were incorporated into the total sample and the analyses were run on one group. However, the number of autistic children in our sample represented the natural variation in the population.
2. Given the Covid-19 pandemic, we have experienced significant difficulties with the recruitment. Instead of 120 typically-developing and 90 children with a clinical profile, a total of 105 children were recruited. For each child, one teacher and one parent participated in the study.
3. Regarding the procedure, the testing sessions with the children took place online through MS Teams instead of Skype for Business. As KU Leuven has, since then, shifted to MS Teams as a default platform for communication, meetings (and testing sessions) due to MS Teams offering end-to-end encryption for 1:1 Teams calls.
4. Regarding the measures, in addition to the measures mentioned and data collected, children's verbal working memory (WM) was assessed. This was done using the Digit Span subtest (forward and backward conditions) of the Wechsler Intelligence Scale for Children – Fifth Edition (WISC-V; Wechsler, 2014). Verbal and visuospatial WM are associated with different cognitive processes: verbal WM is often linked with tasks involving language, such as reading comprehension and vocabulary acquisition, while visuospatial WM is often associated with tasks involving spatial information, such as arithmetic and navigation. Therefore, to obtain a comprehensive view of children's WM, both components ought to be assessed.
5. Furthermore, children's reading abilities were examined using the One-Minute-Test (Een-Minuut- Test; EMT; Brus & Voeten, 1973) and, additionally, Klepel-R (Klepel-Revised; van den Bos, de Groot, & de Vries, 2019). Both tests assess slightly different aspects of reading performance (EMT – word reading fluency, Klepel-R – decoding of non- and pseudo-words speed). Administering these tests together can, therefore, provide a more comprehensive overview of a child's reading abilities. In addition, general academic performance (reported by the teacher) was assessed using a non- standardised short questionnaire asking the teachers to rate children's reading and arithmetic performance on a 5-point Likert scale (from 1 – poor to 5 – excellent). This was opted for given the amount of questionnaires and the time required for the teacher to complete all the questionnaires. Given that the BASC-2 teacher rating scale for school-aged children, contains 160 items, it was decided to use a shorter questionnaire asking teachers to indicate children's abilities more generally.
6. Regarding the statistical analyses, statistical software JASP (JASP Team, 2023, Version 0.17.1.0) was used to analyse the data instead of IBM Statistical Package for the Social Science (SPSS) and MPlus as mentioned in the protocol.

7. No transformations were made if measures were found to be skewed or kurtotic outside the ranges for normally distributed variables. The skewness in some variables (e.g., teacher-student relationship quality) might reflect true nature of this relationship in the population (most participants have an average relationship quality (somewhat close), a few – very good quality (very close), and none – characterised as bad (very conflictual). Therefore, transforming the data might result in a loss of some of this information. Instead, non-parametric alternative (i.e., Spearman's rank correlation) was run, in addition, to Structural Equation Modelling analyses, which are somewhat robust to such violations (provided residuals should be randomly distributed around zero).
8. Regarding missing data, a full information maximum likelihood (FIML) approach was used instead of regression imputation. Given that FIML estimates data based on all the available data, instead of assuming that missing data can be perfectly predicted based on observed data (regression imputation), it is often the preferred method. Furthermore, given that each participant was asked to complete numerous different questionnaires, FIML can handle multiple variables with missing data at once, while regression imputation becomes increasingly complex and potentially less accurate as more variables have missing data.
